# Fingerprints of nonequilibrium stationary distributions in dispersion relations

**DOI:** 10.1038/s41598-021-91455-1

**Published:** 2021-06-08

**Authors:** Kamel Ourabah

**Affiliations:** Theoretical Physics Laboratory, Faculty of Physics, University of Bab-Ezzouar, USTHB, Boite Postale 32, El Alia, Algiers 16111 Algeria

**Keywords:** Plasma physics, Statistical physics, thermodynamics and nonlinear dynamics

## Abstract

Distributions different from those predicted by equilibrium statistical mechanics are commonplace in a number of physical situations, such as plasmas and self-gravitating systems. The best strategy for probing these distributions and unavailing their origins consists in combining theoretical knowledge with experiments, involving both direct and indirect measurements, as those associated with dispersion relations. This paper addresses, in a quite general context, the signature of nonequilibrium distributions in dispersion relations. We consider the very general scenario of distributions corresponding to a superposition of equilibrium distributions, that are well-suited for systems exhibiting only local equilibrium, and discuss the general context of systems obeying the combination of the Schrödinger and Poisson equations, while allowing the Planck’s constant to smoothly go to zero, yielding the classical kinetic regime. Examples of media where this approach is applicable are plasmas, gravitational systems, and optical molasses. We analyse in more depth the case of classical dispersion relations for a pair plasma. We also discuss a possible experimental setup, based on spectroscopic methods, to directly observe these classes of distributions.

## Introduction

Einstein constantly criticized Boltzmann’s statistical approach^1,2^ because, he argues, a statistical description of a system should be ultimately based on its dynamics. This critique opened the way for classical statistics that are different from those of Boltzmann and Gibbs. Since then, compelling evidence has accumulated in favor of the emergence of such non-Boltzmann statistics in a wide spectrum of physical systems and under very general circumstances. In plasma physics and in the physics of gravitational systems, non-Boltzmann distributions are commonplace^3,4^ and are usually understood as a consequence of the long-range nature of the interactions that prevent these systems from reaching thermodynamic equilibrium. Selected examples of systems where such distributions have been observed include driven dissipative dusty plasmas^5^, cold atoms in dissipative optical lattices^6,7^, spin glasses^8^, trapped ions interacting with a classical buffer gas^9^, freestanding graphene membranes^10^, cell monolayer systems^11^, and high energy collisional experiments^12^. A whole sub-branch of physics, dedicated to revealing the dynamical origins of these distributions and to their implementation into different paradigms, is progressively opening up^13–17^.

The most reliable evidence for these distributions comes, of course, from their direct observation. In many situations however, direct observation of distributions can be very cumbersome. In this case, valuable information about the statistical properties of the system can be provided by indirect measurements; that is, by measuring observable quantities that are sensitive to the distribution function. At the forefront of such measurements are those associated with dispersion relations (DRs), which are routinely measured, e.g., in plasma physics or in condensed matter physics, and that carry information on the shape of the distribution. Perhaps the most appealing example in this regard is the measurement of DRs of plasma oscillations provided by Van Hoven^18^ that has been, later on, interpreted as a manifestation of Tsallis statistics^19,20^—a form of non-Boltzmann statistics emerging out of the formalism of nonextensive statistical mechanics (NSM)^21^. It is however important to stress here that, in general, DRs do not contain the full information on the distribution but depend only on its first moments. That is to say, one cannot, from the DRs alone, uniquely identify the distribution. Yet, it is possible to find the signature of a given class of statistics in DRs. It is precisely the aim of this work to attempt to unveil, in a quite general context, the “fingerprints” of nonequilibrium distributions in DRs.

Clearly, DRs depend both on the dynamical (i.e., the equations of motion) and the statistical (i.e., the distribution function) properties of the given system. It is then important to specify the context of our analysis. Let us start with the distributions. To put the discussion on very general grounds, we consider velocity (or energy) distributions in the form of a superposition of local equilibrium distributions, that is

1 $$\begin{aligned} B(v)= \int f(\beta ) f_0(v |\beta ) d \beta , \end{aligned}$$

where $\beta$ is some intensive parameter (hereafter identified with the inverse temperature), which is distributed according to some unspecified distribution $f(\beta )$, while $f_0$ is the (local) equilibrium distribution that can be identified with the Maxwell-Boltzmann (MB) distribution for classical systems or the relevant quantum generalization thereof for a quantum system. Distributions in the form of Eq. (1) are in fact very general; they contain Tsallis distributions as a very special case^22^ but they also cover a variety of other distributions that have been observed experimentally^23–33^. We will focus here on three different distributions $f(\beta )$ (the so-called three universality classes of superstatistics) that have strong empirical evidence. The extension of our analysis to other distributions is nevertheless straightforward conceptually if not technically.

Of no less importance than the statistics is, of course, the dynamics of the system. To make the discussion as general as possible, we will be considering systems that can be formally described by the combination of the Schrödinger and the Poisson equations (SP model in short), while allowing the Planck’s constant to smoothly go to zero, yielding the classical Vlasov-Poisson regime. Typical systems obeying the SP model are quantum plasmas^34,35^ and self-gravitating systems^36–38^. However, as pointed out recently^39^, the applicability of the SP model goes well beyond the usual scenario of plasmas and self-gravitating systems and covers a large spectrum of systems, manifesting similar elementary excitations, such as Bose-Einstein condensates (BECs) and optical molasses.

This paper progresses in the following fashion. First, we discuss the velocity distributions (classical and quantum) in the form of Eq. (1), corresponding to the three universality classes of superstatistics. To show their significance, we confront them with recent observations of relevance for our discussion, i.e., those of space plasmas in the presence of micro-gravity^40^. Next, we work out the corresponding DRs in the context of the SP model and the corresponding Vlasov-Poisson limit. We examine more closely the classical limit of these DRs in the case of a pair (electron-positron) plasma, discussing the different modes and the corresponding Landau damping. Finally, we present an experimental setup, based on spectroscopic methods, to directly observe these distributions.

## Nonequilibrium stationary distributions

To set the scene, let us first state precisely the statistical conditions we are considering here, i.e., the circumstances under which Eq. (1) holds. We are dealing, quite generally, with nonequilibrium systems in a stationary state that exhibit only *local equilibrium*. In this situation, our nonequilibrium system may be virtually divided up into small cells or small regions that remain infinitely close to equilibrium. In each cell, one has local thermal equilibrium and the (local) statistics $f_0(v |\beta )$ correspond to those of equilibrium statistical mechanics (in fact, as each cell exhibits local equilibrium, the whole machinery of equilibrium statistical mechanics holds locally). At larger scales however, one has to account for temperature inhomogeneities across different cells, that are encoded into $f(\beta )$. Provided that the temperature varies within a time-scale much larger than the local relaxation time (viz., the adiabatic *Ansatz*^41^), the long-term distribution arises as a superposition of local equilibrium statistics, averaged over the distribution of the inverse temperature $\beta \equiv 1/k_B T$ (hereafter, $k_B=1$) across the different cells, i.e., Eq. (1). In the statistical mechanics literature, such an approach goes by the name of *superstatistics*^22^, as it merely consists of a superposition of different statistics. As we will discuss later on, the conditions outlined above are in fact very common and this methodological attitude allows describing a wide range of physical systems under very typical conditions.

In principle, one may construct infinitely many distributions in the form of Eq. (1), by selecting appropriate distributions $f(\beta )$. It is known however that three main classes of $f(\beta )$ emerge as a universal behavior for most experimentally relevant situations. Beside their experimental relevance, these universality classes have a transparent statistical origin that can be understood from the application of the central limit theorem^23,24^ or from the maximum entropy principle^42^ (see also Ref.^43^). Assuming a (*d*-dimensional) MB distribution for $f_0(v |\beta )$, we present in what follows these classes and the corresponding long-term velocity distributions.

**Figure 1:**
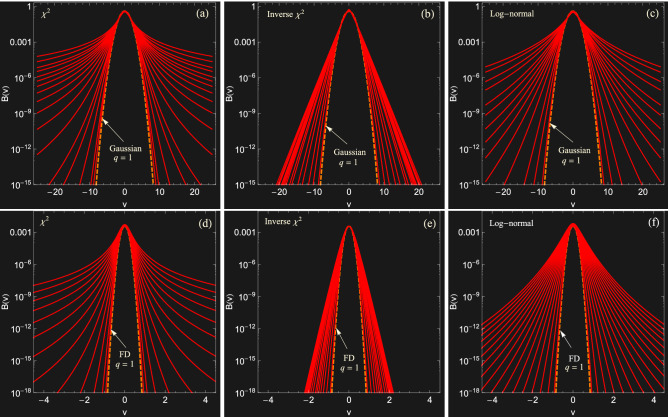
Examples of superstatistical velocity distributions for $\chi ^2$ (**a**, **d**), inverse-$\chi ^2$ (**b**, **e**), and log-normal (**c**, **f**) superstatistics, with different values of $q:=\langle \beta ^2 \rangle / \beta _0^2$, in a logarithmic scale to better highlight the tails. In the top panel, the local equilibrium distribution corresponds to the MB distribution while in the bottom panel, it corresponds to the (1d projected) FD distribution.

(i) $\chi ^2$ *superstatistics* In this case, the inverse temperature $\beta$ is distributed according to the $\chi ^2$ distribution (also known as Gamma distribution) of degree *n*:

2 $$\begin{aligned} f(\beta )=\frac{1}{\Gamma \left( \frac{n}{2}\right) }\left( \frac{n}{2 \beta _{0}}\right) ^{n / 2} \beta ^{n / 2-1} e^{-\frac{n \beta }{2 \beta _{0}}}, \end{aligned}$$

where $\beta _0 \equiv \langle \beta \rangle$ is the average of $\beta$. The corresponding long-term velocity distribution follows from Eq. (1) as

3 $$\begin{aligned} B(v)= \int _0^{\infty } d \beta f(\beta ) \left( {\frac{\beta m}{2 \pi }}\right) ^{d/2} \exp \Big [-\frac{\beta m v^{2}}{2}\Big ] = \left( \frac{\beta _0 m}{\pi n} \right) ^{d/2} \frac{ \Gamma \left( \frac{n+d}{2}\right) }{ \Gamma \left( \frac{n}{2}\right) }\left( 1+\frac{\beta _{0}}{n}m v^{2}\right) ^{-\frac{n+d}{2}}, \end{aligned}$$

which is equivalent to the Tsallis distribution (*q*-Gaussian), known in the paradigm of NSM. This can be made more transparent by adopting a slightly different parametrization; by defining an entropic index ${\widetilde{q}} := 1+ 2/(n+d)$ and an effective inverse temperature ${\widetilde{\beta }} :=\beta _0 (n+d) /n \equiv 1/{\widetilde{T}}$, Eq. (3) can be re-expressed in the more familiar form^21^ as

4 $$\begin{aligned} B(v) = \frac{\Gamma [\frac{1}{{\widetilde{q}}-1}]}{\Gamma [\frac{1}{{\widetilde{q}}-1}-\frac{1}{2}]}\sqrt{\frac{m({\widetilde{q}}-1)}{2 \pi {\widetilde{T}}}} \left[ 1+\left( {\widetilde{q}} -1\right) {\widetilde{\beta }} \frac{ m v^2}{2} \right] ^{\frac{1}{1-{\widetilde{q}} }}. \end{aligned}$$

We note in passing that, in the statistics literature, distributions in the form of Eqs. (3)–(4) are known as Student’s t-distributions^44^. Note that for large *v*, these distributions behave asymptotically as power-laws, which make them relevant for many different physical problems.

(ii) *Inverse*$\chi ^2$* superstatistics* In this case, instead of $\beta$, it is the temperature ($\beta ^{-1}$) itself that is $\chi ^2$-distributed. Then, $\beta$ follows an inverse-$\chi ^2$ distribution,

5 $$\begin{aligned} f(\beta )=\frac{\beta _{0}}{\Gamma \left( \frac{n}{2}\right) }\left( \frac{n \beta _{0}}{2}\right) ^{n / 2} \beta ^{-n / 2-2} e^{-\frac{n \beta _{0}}{2 \beta }}. \end{aligned}$$

The corresponding velocity distribution follows as

6 $$\begin{aligned} B(v)= \frac{2\beta _0}{ \Gamma (\frac{n}{2})} \left( \frac{m}{2 \pi }\right) ^{d/2} \left( \frac{\beta _0 n}{2} \right) ^{n/2} \left( \frac{m v^2}{\beta _0 n} \right) ^{\frac{2-d+n}{4}} {\mathcal {K}}_{\frac{2-d+n}{2}}(\sqrt{n m \beta _{0}}|v|), \end{aligned}$$

where ${\mathcal {K}}_{\alpha }(x)$ is the modified Bessel function of the second kind. Asymptotically, these distributions exhibit exponential tails in the velocity^45^, which make them of less relevance in the usual scenario of space plasmas and gravitating system that are usually characterized by heavy-tailed distributions. Nonetheless, this type of exponential behavior has been observed for stationary distributions in the case of Vortex glasses and vortex liquids^46^, in fusion plasmas^27^, and in cancer disease-specific mortality probability distributions^28^.

(iii) *Log-normal superstatistics* In this case, $\beta$ is distributed according to the log-normal distribution,

7 $$\begin{aligned} f(\beta )=\frac{1}{\sqrt{2 \pi } s \beta } \exp \left\{ \frac{-\left( \ln \frac{\beta }{\mu }\right) ^{2}}{2 s^{2}}\right\} , \end{aligned}$$

with an average of $\beta$ given by $\beta _0 \equiv \langle \beta \rangle =\mu e^{s^2/2}$. In this case, no closed-form expression for the corresponding velocity distribution *B*(*v*) is known to date. Therefore, we will be dealing with this last case numerically.

These three classes of distributions cover the rich variety of distributions observed in stationary nonequilibrium systems and, in fact, have substantial empirical evidence; $\chi ^2$ superstatistics (or equivalently Tsallis statistics) have been observed in numerous systems, such as dusty plasmas^5^, cold atoms^6,7^, and spin glasses^8^. Experimental evidence for log-normal superstatistics has been reported in the context of Lagrangian and Eulerian turbulence^25,26^, gravitational systems^38^, space plasmas^47^, and air pollution statistics^33^, among other systems^48^, while systems possibly obeying inverse-$\chi ^2$ superstatistics have been presented in Refs.^27–29^.

So far, we have been considering the case of classical statistics. One may nonetheless follow similar lines of reasoning in the quantum context as well by identifying the local equilibrium distribution $f_0(v |\beta )$ to the Bose–Einstein (BE) or Fermi–Dirac (FD) distribution. The latter case is particularly interesting for our analysis, as we will focus our attention on plasma DRs. In this case, the local equilibrium FD velocity distribution reads as

8 $$\begin{aligned} f_0(v |\beta ) \propto \frac{1}{1+e^{\beta (\epsilon -\mu )}}, \end{aligned}$$

where $\epsilon =m v^{2} / 2$. At this stage, an important remark is in order. Note that, although phenomena of wave propagation, producing DRs, are intrinsically 1d processes, the distribution is fundamentally a 3d quantity. In the classical case of the MB distribution, this subtlety can be safely disregarded since the 1d MB distribution, obtained by integrating over the two directions perpendicular to the wave propagation, has the same form as the original 3d distribution. In the case of the FD distribution, the reduction of the 3d distribution to its projected 1d version has to be done more carefully, and reads as^49^

9 $$\begin{aligned} f_{0}^{(1d)}\left( v |\beta \right) =\frac{3}{4 v_{F}} \frac{\beta _F}{\beta } \ln \left[ 1+\exp \left\{ - \beta \left( {\frac{1}{2} m v^{2}-\mu } \right) \right\} \right] , \end{aligned}$$

where $\beta _F \equiv 1/T_F$, with $T_F$ being the Fermi temperature and $v_F$ the Fermi velocity. In this case, we are not in position to obtain closed-form expressions for the corresponding superstatistical distributions (1). The latter can however be easily computed numerically.

In Fig. 1, we show examples of 1d velocity distributions associated with the three universality classes of superstatistics, i.e., $\chi ^2$ [Eq. (2)], inverse $\chi ^2$ [Eq. (5)], and log-normal [Eq. (7)]. The top panel corresponds to a local equilibrium MB distribution while the bottom panel shows its generalization to the FD distribution (for $T=0.01 T_F$ and $\mu =0$). For a better comparison between the different universality classes, we parametrize the distributions by using a single parameter defined as $q:= \langle \beta ^2 \rangle / \beta _0^2$. The latter can be easily expressed in terms of the parameters of the corresponding distribution $f(\beta )$ as

10 $$\begin{aligned} \begin{aligned} q&:= \frac{\langle \beta ^2\rangle _{\chi ^2}}{\beta _0^2}= 1+\frac{2}{n} \quad (n>2), \\ q&:= \frac{\langle \beta ^2\rangle _{inv. \chi ^2}}{\beta _0^2}= \frac{n}{n-2}, \\ q&:= \frac{\langle \beta ^2\rangle _{LN}}{\beta _0^2}= e^{s^2}, \end{aligned} \end{aligned}$$

for the three universality classes. This parametrization is convenient since the limit $q=1$ corresponds to a vanishing variance of $f(\beta )$, which shrinks into a Dirac delta $\delta (\beta - \beta _0)$, and equilibrium distributions are recovered in this case.

**Figure 2:**
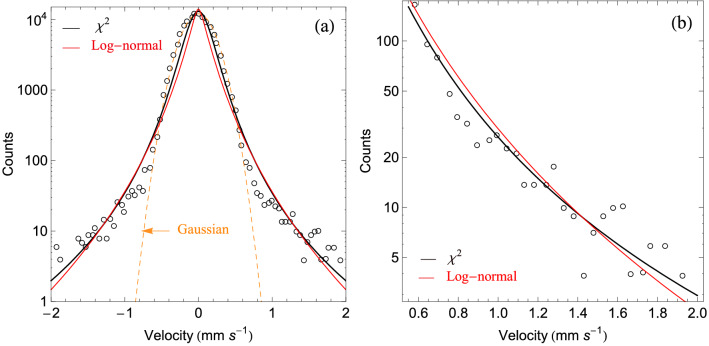
Superstatistical distribution functions corresponding to the $\chi ^2$ (black) and log-normal (red) universality classes, compared to the data of Ref.^40^ (open circles), obtained from the analysis of a time series using the Complex Plasma Laboratory PK-4 instrument on-board the ISS. The left panel (**a**) shows the whole domain while the right panel (**b**) shows only the halo part, i.e, the high energy tail.

Before closing this section, it may be appropriate to give these distributions a more empirical credit, by confronting them with direct observations, in a context relevant to our analysis. In Fig. 2a, we confront them with independent observations^40^ of velocity distributions of a plasma under micro-gravity conditions, obtained through the PK-4 instrument on-board the International Space Station (ISS), that clearly exhibit a non-Boltzmann behavior. We show the best-fits obtained by a nonlinear regression method based on the Levenberg-Marquardt algorithm^50,51^ (also known as the damped least-squares method), for the $\chi ^2$ and the log-normal universality classes, while we disregard the inverse-$\chi ^2$ class, which fails to describe the high-energy part of the observational data, since it exhibits an exponential decay. One may appreciate that both the $\chi ^2$ and the log-normal classes nicely fit the data. Note that an even better fit can be reached by considering (as usually done in reports) that the core population is described by a MB distribution, and using the superstatistical distributions to fit only the halo part, i.e., the high energy tails, as shown in Fig. 2b.

## Generic dispersion relations: the Schrödinger–Poisson system

To put the discussion in a very general context, we consider here generic dispersion relations that apply to a wide class of systems, formally described by the combination of the Schrödinger equation and the Poisson equation. As a trivial example, one may think of the case of a quantum plasma, where the SP model reads as

11 $$\begin{aligned} \begin{aligned} \mathrm {i} \hbar \frac{\partial \psi }{\partial t}&=\left( -\frac{\hbar ^{2}}{2 m} \nabla ^{2}-e \Phi _{C}\right) \psi , \\ \nabla ^{2} \Phi _{C}&= \frac{e}{\varepsilon _0} (|\psi |^{2}-n_0), \end{aligned} \end{aligned}$$

where $\Phi _{C}$ is the Coulombian potential and $n_0$ is the background ion density (with ions supposed at rest). Another typical example is that of a self-gravitating system, in which case one has

12 $$\begin{aligned} \begin{aligned} \mathrm {i} \hbar \frac{\partial \psi }{\partial t}&=\left( -\frac{\hbar ^{2}}{2 m} \nabla ^{2}+m \Phi _{G}\right) \psi , \\ \nabla ^{2} \Phi _{G}&=4 \pi m G|\psi |^{2}, \end{aligned} \end{aligned}$$

where $\Phi _G$ is the gravitational potential and *m* is the mass of the particle. Such a formal analogy between plasmas and self-gravitataing systems is well-known and, indeed, is also manifest in the classical regime, i.e., in the Vlasov–Poisson kinetic approach. However, as recently observed by Mendonça^39^, this analogy goes even further and applies to other physical problems as well. This can be made more transparent by rewriting the SP model in the form of an integro-differential equation as

13 $$\begin{aligned} \mathrm {i} \hbar \frac{\partial \psi }{\partial t}=\left[ -\frac{\hbar ^{2}}{2 m} \nabla ^{2}+\Phi _{0}+g \int {\mathcal {U}}\left( \mathrm {r}-\mathrm {r}^{\prime }\right) \left| \psi \left( \mathrm {r}^{\prime }, t\right) \right| ^{2} \mathrm {dr}^{\prime }\right] \psi , \end{aligned}$$

where $\Phi _0$ is an arbitrary external potential. Upon choosing the functional form ${\mathcal {U}}\left( \mathrm {r}-\mathrm {r}^{\prime }\right) ={1}/{\left| \mathrm {r}-\mathrm {r}^{\prime }\right| }$, one covers the case of (i) a quantum plasma ($g={e^{2}}/{4 \pi \varepsilon _{0}}$ and $\Phi _{0}=-{e^{2}}/{\varepsilon _{0}} n_{0}$), (ii) a self-gravitating system ($g=-m^{2} G$ and $\Phi _0=0$), and (iii) ultra-cold atoms in a magneto-optical trap ($g={Q}/{4 \pi }$ and $\Phi _{0}=-Q n_{\mathrm {eq}}$, where *Q* is the effective atomic charge and $n_{eq}$ is the equilibrium density). The model also covers (iv) the case of a BEC, with or without dipolar interactions, for

14 $$\begin{aligned} {\mathcal {U}}\left( {\mathbf {r}}-{\mathbf {r}}^{\prime }\right) =\delta \left( {\mathbf {r}}-{\mathbf {r}}^{\prime }\right) +\frac{C_{d d}}{8 \pi g} \frac{1-3 \cos ^{2} \theta }{\left| {\mathbf {r}}-{\mathbf {r}}^{\prime }\right| ^{3}}\left( 3 \cos ^{2} \varphi -1\right) \end{aligned}$$

and $g=4 \pi \hbar ^{2} a m$, where *a* is the scattering length, $C_{dd}$ is the dipolar interaction strength while $\theta$ and $\varphi$ are orientation angles. By employing the quantum kinetic Wigner-Moyal approach^52,53^, one arrives at a generic form of the DR as follows

15 $$\begin{aligned} 1-\frac{g n_{0} k^{2}}{m \omega ^{2}} {\mathcal {U}}(\mathrm {k}) \int \frac{f_{0}(v) \mathrm {d} v}{(1-k v / \omega )^{2}-\hbar ^{2} k^{4} / 4 m^{2} \omega ^{2}}=0, \end{aligned}$$

where $f_0(v)$ is the unperturbed (1d projected) velocity distribution and ${\mathcal {U}}(\mathrm {k})$ is the Fourier transform of ${\mathcal {U}}\left( \mathrm {r}-\mathrm {r}^{\prime }\right)$ (for plasmas, self-gravitating systems, and ultra-cold atoms, one has ${\mathcal {U}}(\mathrm {k})=4\pi /\mathrm {k}^2$). For a system, initially in thermodynamic equilibrium, $f_0(v)$ is usually identified with the MB distribution, or the relevant quantum generalization thereof. For a system exhibiting only local equilibrium, as defined above, the unperturbed distribution $f_0(v)$ takes the form of Eq. (1), where the temperature distribution $f(\beta )$ is given by one of the three universality classes. While computing DRs, one is most often interested in the case of excitations with large phase velocities, i.e., $v \ll \omega / k$. In this limit, Eq. (15) can be expanded as follows

16 $$\begin{aligned} \omega ^{2}=\omega _0^{2}\left( 1+ 3\frac{k^{2}}{\omega ^{2}}\left\langle v^{2}\right\rangle +\frac{\hbar ^{2} k^{4}}{4 m^{2} \omega ^2} + 5\frac{k^{4}}{\omega ^{4}}\left\langle v^{4}\right\rangle + \ldots \right) , \end{aligned}$$

where $\omega _0$ is the appropriate characteristic frequency, given by

17 $$\begin{aligned} \omega _0^{2}=\frac{g n_{0}}{m} k^{2} {\mathcal {U}}({\mathbf {k}}). \end{aligned}$$

For a quantum plasma, it corresponds to the plasma frequency $\omega _0 \equiv \omega _p=\sqrt{e^2 n_0/\varepsilon _0 m}$, while for a self-gravitating system it is given by $\omega _0^2 \equiv -\omega _J^2$, where $\omega _J=\sqrt{4 \pi G m n_0}$ is the so-called Jeans frequency.

Note that, in principle, DRs (16) fully characterize the velocity distribution through the complete (infinite) set of velocity moments $\langle v^l \rangle$. In practice however, one is interested in the experimentally relevant limit of excitations with large phase velocities, in which case Eq. (16) is truncated and contains information on the velocity distribution only through its first moments. That is, one cannot reconstruct the distribution function from the DR alone. This turns out to be rather advantageous if one is interested in the universal effect produced by nonequilibrium distributions in DRs. This is because, even in the absence of a closed-form expression for the distribution, as happens in the case of log-normal superstatistics, one can compute the corresponding DR as long as the moments of $f(\beta )$ are known. In fact, one may observe that, for distributions in the form of Eq. (1), the velocity moments can be expressed as a combination of the moments of the local equilibrium distribution $f_0(v |\beta )$ with those of $f(\beta )$ as follows

18 $$\begin{aligned} { \langle {v}^{l} \rangle } \equiv { \int {v}^{l} B(v) d {v}}= \langle \langle {v}^{l} \rangle _{f_0}\rangle _{f(\beta )}, \end{aligned}$$

where $\langle \cdot \rangle _{f_0}$ stands for an average over the equilibrium distribution $f_0(v |\beta )$ and $\langle \cdot \rangle _{f(\beta )}$ is an average over $f(\beta )$. For the three universality classes, i.e., Eqs. (2), (5), and (7), one has

19 $$\begin{aligned} \begin{aligned} \langle \beta ^l\rangle _{\chi ^2}&=\frac{\Gamma \left( \frac{n}{2}+l\right) }{\Gamma \left( \frac{n}{2}\right) }\left( \frac{2}{n}\right) ^{l}\beta _0^l,\\ \langle \beta ^l\rangle _{inv. \chi ^2}&=\frac{\Gamma \left( \frac{n}{2}+1-l\right) }{\Gamma \left( \frac{n}{2}\right) }\left( \frac{n}{2}\right) ^{l-1}\beta _0^l,\\ \langle \beta ^l\rangle _{LN}&= e^{ {l(l-1)s^2}/{2}} \beta _0^l, \end{aligned} \end{aligned}$$

that can be combined with those of the (1d) MB distribution

20 $$\begin{aligned} \langle v^l\rangle _{MB}= \frac{ (l-1)!!}{(\beta m)^{l/2}} \end{aligned}$$

(*l* even), to determine all superstatistical velocity moments in an exact fashion. In particular, the first two moments appearing in the DR (16) follow as

21 $$\begin{aligned} \begin{aligned} \langle v^2 \rangle _{i}&= \phi _i(q) \langle v^2 \rangle , \\ \langle v^4 \rangle _{i}&= \gamma _i(q) \langle v^4 \rangle , \end{aligned} \qquad (i=1,2,3) \end{aligned}$$

where the auxiliary functions $\phi _i(q)$ and $\gamma _i(q)$ read as

22 $$\begin{aligned} \begin{aligned} \phi _1(q)&\equiv \frac{1}{2-q} \quad (1<q<2),\\ \phi _2(q)&\equiv \frac{2q-1}{q}, \quad \\ \phi _3(q)&\equiv q, \end{aligned} \end{aligned}$$

and

23 $$\begin{aligned} \begin{aligned} \gamma _1(q)&\equiv \frac{1}{6+q(2q-7)} \quad (1<q<3/2),\\ \gamma _2(q)&\equiv 6+ \frac{2-7q}{q^2}, \quad \\ \gamma _3(q)&\equiv q^3. \end{aligned} \end{aligned}$$

Above, $i=1,2,3$ stand for $\chi ^2$, inverse-$\chi ^2$, and log-normal superstatistics respectively. We can convince ourselves that nonequilibrium distributions have indeed a universal effect on DRs by observing that,by construction, one has $q:=\langle \beta ^2 \rangle / \beta _0^2 \ge 1$, which implies that $\phi _i(q) \ge 1$ and $\gamma _i(q) \ge 1$, where the equality holds at equilibrium (i.e., for $q=1$). The effect of nonequilibrium distributions is that of increasing the velocity moments, that are larger than their equilibrium counterparts; a feature that may be attributed to the heavy tails that are typical of nonequilibrium stationary distributions.

Note that in the case of a local equilibrium distribution given by the FD distribution, the moments are difficult to obtain in an exact form. One may use however approximate expressions^54,55^ that are in agreement with the exact forms that are known in the dilute (classical) and ultra-dense (completely degenerate) cases. For instance, for the kinetic energy (i.e., the second-order velocity moment), one usually employs the arithmetic sum of the MB thermal energy and the Fermi energy. Hence, as far as this approximation is valid, the above discussion is applicable to the FD case as well.

**Figure 3:**
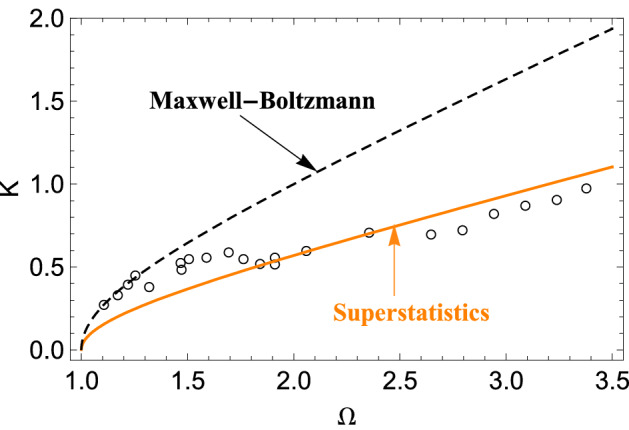
Comparison between the experimental data of Ref.^56^ (open circles) and the best-fit obtained using the generic DR (25), corresponding to $\phi _i(q) \approx 3.08$ (solid line). The equilibrium case $q=1$ is also shown for comparison (dashed line).

Note that the classical limit of the DR (15) is easily obtained by letting $\hbar \rightarrow 0$, and reduces to

24 $$\begin{aligned} 1+{g n_0} {\mathcal {U}}(\mathrm {k}) k \int \frac{\partial f_{0}/ \partial v}{(\omega -k v)} dv=0, \end{aligned}$$

which coincides with the classical DR corresponding to the the Vlasov–Poisson kinetic treatment. In particular, in the case of a plasma, by considering the large phase velocity limit ($v \ll \omega / k$) and neglecting terms beyond the order $k^2$, one obtains after making use of Eq. (21), the following DR

25 $$\begin{aligned} \omega ^{2}=\omega _{0}^{2}\left[ 1+3 \phi _i(q) \left( k \lambda _{D}\right) ^{2} \right] , \end{aligned}$$

which is a modified version of the Bohm-Gross DR that have been already discussed in^47^, where $\lambda _D \equiv \sqrt{\varepsilon _0 T_0/ n_0 e^2}$ is the Debye length defined at the mean temperature $T_0$. In Ref.^47^, we have shown that DRs in the form of Eq. (25) fit nicely with the data of Van Hoven^18^ that have been argued to be a manifestation of Tsallis statistics^19,20^. Here, we compare the DR (25) with the dispersion data of Derfler *et al.*^56^, obtained using a Langmuir probe. Best-fits obtained using the generic DR (25) are shown in Fig. 3, where we have normalized the frequency to $\omega _0$ and the wavenumber to $1/ \lambda _D$, that is, $\Omega \equiv \omega / \omega _0$ and $K \equiv \lambda _D k$. One may appreciate that the universal effect induced by nonequilibrium distributions is in a good agreement with the data.

Prior to analyzing more general forms of DRs in the following section, it may be appropriate to comment briefly on the effect of nonequilibrium stationary distributions on the coupling parameter. In a plasma, the latter is defined as the ratio between the average electrostatic potential per particle $\langle U \rangle$ and the average kinetic energy $\langle K \rangle$. Independently on the distribution, one can estimate $\langle U\rangle \approx e^{2} /4 \pi \varepsilon _0 r_{S}$ where $r_s$ is the Wigner-Seitz radius defined as $4 \pi r_{S}^{3}n_{0} / 3 =1$. On the other hand, for a nonequilibrium distribution in the form of (1), one has $\langle K\rangle =m \langle v^2 \rangle /2 =3 \phi _i(q)T_0/m$. The coupling parameter follows as

26 $$\begin{aligned} \Gamma ^{(q)} \equiv \frac{\langle U\rangle }{\langle K\rangle }=\frac{e^2}{6 \varepsilon _0} \left( \frac{4}{3 \pi ^2} \right) ^{1/3} \frac{n_0^{1/3}}{\phi _i(q)T_0} \le \Gamma ^{(q=1)}. \end{aligned}$$

That is, a plasma in a nonequilibrium stationary state tends to be less coupled than its equilibrium counterpart (at the same mean temperature); a feature that may be attributed to the haivy tails of the nonequilibrium distributions, i.e., to the high energy population.

## Classical dispersion relations: the case of a pair plasma

In this section, we examine in further details more general forms of classical DR (24), and the corresponding Landau damping, in the case of a plasma. Instead of an electron-ion plasma, we consider the case of a pair plasma, composed of electrons and positrons (or equivalently, two ion species having the same mass^57^). In fact, because of the mass symmetry and the charge anti-symmetry between the two species, electrons and positrons in general follow (apart from a small asymmetry) the same distribution with the same mean temperature. It is therefore meaningful to assign the same superstatistical distribution to both species. The two-species extension of the DR (24) reads as

27 $$\begin{aligned} 1-\frac{\omega _0^{2}}{ k^{2}} \int \frac{\frac{\partial }{\partial v}\left( f_{-}(v)+f_{+}(v)\right) }{v-\frac{\omega }{k}} \mathrm {~d} v=0, \end{aligned}$$

where the subscripts (−) and (+) stand for electrons and positrons, respectively, and $\omega _0$ is the plasma frequency for a pair plasma, $\omega _0= \sqrt{2 n_0 e^2/ \varepsilon _0 m}$.

Let us first analyze, as previously, the high frequency limit ($v \ll \omega / k$) of the DR (27), i.e., Langmuir modes. In this case, by considering only the real part of the DR for the moment, we recover the modified Gross-Bohm relation (25), where the Debye length is modified accordingly for a pair plasma, i.e., $\lambda _D \equiv \sqrt{\varepsilon _0 T_0 / 2 n_0 e^2}$. Note however that, because of to the singularity in the integrand of Eq. (27), the integral is not properly defined in the whole velocity domain. In fact, this singularity induces an imaginary part in the DR, which is responsible for the Landau damping. To study this process, we follow very standard lines (see for instance Ref.^58^), by setting $\omega =\omega _r+ i \gamma$, and restricting ourselves to a small imaginary part, i.e., $\vert \gamma \vert \ll \omega _r$. It is convenient to re-express the DR (27) as $D(k, \omega )=0$ and split it into a real and imaginary parts, i.e., $D=D_r + i D_i$, where $D_r$ is determined by the Cauchy principal value of the integral, while for $D_i$, one retains the pole contribution to the integral. Then, the imaginary part of the frequency is given as^58^

28 $$\begin{aligned} \gamma =-\frac{D_{i}\left( k, \omega _{r}\right) }{\partial D_{r}\left( k, \omega _{r}\right) / \partial \omega _{r}}. \end{aligned}$$

Following these lines, we have for the $\chi ^2$ and inverse-$\chi ^2$ classes:

29 $$\begin{aligned} \gamma =-\sqrt{\frac{\pi }{8}} \frac{\Gamma \left[ \frac{3}{2}+\frac{1}{q-1}\right] }{\Gamma \left[ \frac{1}{q-1}\right] } \frac{(q-1)^{3 / 2} \omega _{0}}{\left( \lambda _{D} k\right) ^{3}}\left[ 1+\frac{(q-1)}{2 \lambda _{D}^{2} k^{2}}\right] ^{\frac{1}{1-q}-\frac{3}{2}}, \end{aligned}$$

and

30 $$\begin{aligned} \gamma =-\sqrt{\frac{\pi }{8}} \frac{2^{\frac{q-3}{4 q-1}+\frac{1}{2}}}{\Gamma \left[ \frac{q}{q-1}\right] }\left( \frac{q}{q-1}\right) ^{\frac{q}{q-1}}\left[ \frac{q-1}{q \lambda _{D}^{2} k^{2}}\right] ^{\frac{1+q}{4 q-1}} \frac{\omega _{0}}{\lambda _{D}^{3} k^{3}} {\mathcal {K}}_{\frac{q+1}{2(q-1)}}\left( \sqrt{\frac{2 q}{(q-1) \lambda _{D}^{2} k^{2}}}\right) , \end{aligned}$$

whereas for the log-normal class, we are not in position to obtain a closed-form expression for $\gamma$. The latter can however be expressed in the following integral form

31 $$\begin{aligned} \gamma =-\sqrt{\frac{\pi }{8}} \int _{0}^{\infty } d \beta \frac{(\beta m)^{3 / 2} \omega _{0}^{4}}{\sqrt{2 \pi \ln (q)} \beta k^{3}} e^{-\frac{[\ln (\beta / \sqrt{q})]^{2}}{2 \ln (q)}-\frac{\beta m \omega _{0}^{2}}{2 k^{2}}}, \end{aligned}$$

which can be easily solved numerically. In addition to Langmuir modes, corresponding to the high frequency branch, one can also investigate the low frequency branch or ion-acoustic waves (IAWs). In this case, we employ the usual trick^59^ that consists of assuming a difference in the (mean) temperatures of the two species, i.e., $T_{+} \lesssim T_{-}$ and considering a phase velocity such that $v_{t h,+} \lesssim {\omega }/{k} \lesssim v_{t h,-}$, where $v_{t h,\pm }$ are the thermal speeds of the two species. In the end of the process, we let $T_+ \rightarrow T_{-}=T_0$. In this case, from the real part of the DR (27), we obtain

32 $$\begin{aligned} \omega _r^{2}=k^{2} \lambda _D^{2}\left[ \frac{1}{2\left( k \lambda _{D}\right) ^{2}+1}+3 \phi _i(q) \right] \omega _0^2, \end{aligned}$$

where we use the subscript *r* to indicate that it corresponds to the real part of the DR. From the imaginary part, we obtain for $\chi ^2$ and inverse-$\chi ^2$, the following expressions

33 $$\begin{aligned} \gamma =-\sqrt{\frac{\pi }{2}} \frac{\Gamma \left[ \frac{3}{2}+\frac{1}{q-1}\right] }{\Gamma \left[ \frac{1}{q-1}\right] } (q-1)^{3 / 2} \omega _{r} \left( \frac{1}{2\left( k \lambda _{D}\right) ^{2}+1}+ \frac{3}{2-q} \right) ^{3/2} \times \left[ 1+\frac{(q-1)}{2 } \left( \frac{1}{2\left( k \lambda _{D}\right) ^{2}+1}+\frac{3}{2-q} \right) \right] ^{\frac{1}{1-q}-\frac{3}{2}} \end{aligned}$$

and

34 $$\begin{aligned} \begin{aligned} \gamma =&-\sqrt{\frac{\pi }{2}} \frac{2^{\frac{q-3}{4 q-1}+\frac{1}{2}}}{\Gamma \left[ \frac{q}{q-1}\right] }\left( \frac{q}{q-1}\right) ^{\frac{q}{q-1}} \left( \frac{1}{2\left( k \lambda _{D}\right) ^{2}+1}+ \frac{6q-3}{q} \right) ^{3/2} {\omega _{r}} \left[ \frac{(q-1) \left( \frac{2}{2\left( k \lambda _{D}\right) ^{2}+1}+ \frac{12q-6}{q} \right) {}}{q }\right] ^{\frac{1+q}{4 q-1}} \\&\times {\mathcal {K}}_{\frac{q+1}{2(q-1)}}\left( \sqrt{\frac{4 q \left( \frac{1}{2\left( k \lambda _{D}\right) ^{2}+1}+ \frac{6q-3}{q} \right) {}}{(q-1) }}\right) , \end{aligned} \end{aligned}$$

respectively, whereas for the class of log-normal superstatistics, we have the following integral form

35 $$\begin{aligned} \gamma =-\sqrt{\frac{\pi }{2}} {\omega _r}\left( \frac{1}{2\left( k \lambda _{D}\right) ^{2}+1}+3 q \right) ^{3/2} \int _{0}^{\infty } d \beta \frac{(\beta / \beta _0 )^{3 / 2} }{\sqrt{2 \pi \ln (q)} \beta } e^{-\frac{[\ln (\beta / \sqrt{q})]^{2}}{2 \ln (q)}-\frac{\beta }{2 \beta _0} \left( \frac{1}{2\left( k \lambda _{D}\right) ^{2}+1}+3 q \right) }. \end{aligned}$$

**Figure 4:**
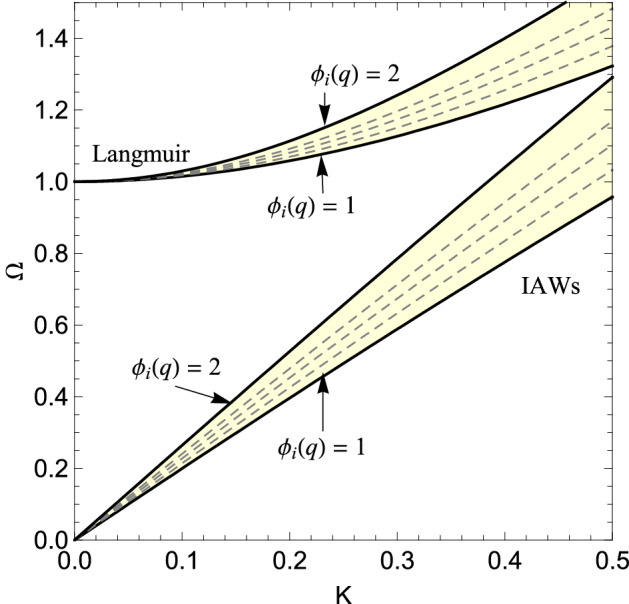
Brillouin diagrams corresponding to the generic DRs for Languir modes [Eq. (25)] and IAWs [Eq. (32)], for different values of the auxiliary function $\phi _i(q)$. The corresponding degree of inhomogeneities, i.e., $q:=\langle \beta ^2 \rangle / \beta _0^2$, can be obtained, for each class of superstatistics, by inverting Eq. (22).

At this stage, it is instructive to observe that the real part of the DR depends on the distribution only through the auxiliary function $\phi _i(q)$. This is no longer true for the imaginary part, i.e., for damped modes. In Fig. 4, we show the real part of the DRs (Brillouin diagrams) for the case of Langmuir modes [Eq. (25)] and for IAWs [Eq. (32)], with different values of the auxiliary function $\phi _i(q)$. One may see that, indeed, the effect of different nonequilibrium distributions on the DR is qualitatively the same. By inverting Eq. (22), one can deduce, for the different universality classes, the corresponding value of $q:= \langle \beta ^2 \rangle / \beta _0^2$, which measures the degree of temperature inhomogeneities.

**Figure 5:**
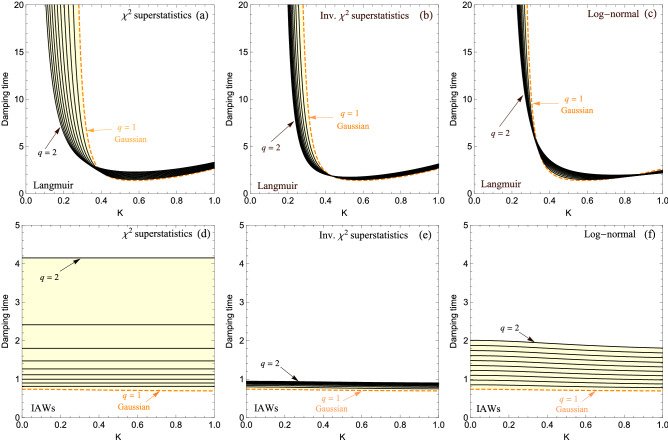
Normalized Landau time for the three universality classes of superstatistics: $\chi ^2$ (**a**, **d**), inverse-$\chi ^2$ (**b**, **e**), and log-normal (**c**, **f**) superstatistics. The top panel corresponds to Langmuir modes, i.e. Eqs. (29)–(31), whereas the bottom panel corresponds to IAWs, i.e., Eqs. (33)–(35).

The imaginary part of the DR is, on the contrary, more sensitive to the distribution. In Fig. 5, we show the Landau time, i.e., $1/\vert \gamma \vert$, normalized to $1/\omega _r$, for Langmuir modes [Eqs. (29)–(31)] and for IAWs [Eqs. (33)–(35)], for the three universality classes of superstatistics. Although one sees the same tendency for the three universality classes, it appears that one can, at least in principle, distinguish between them. In practice however, this may be a highly nontrivial matter, inasmuch as the three universality classes induce qualitatively the same effect.

Before closing this section, it should be noted that beside the two modes discussed above, which are longitudinal modes, one also has transverse electromagnetic waves or light waves. In this case, the classical kinetic treatment yields^59^

36 $$\begin{aligned} \omega ^{2}=k^{2} c^{2}+ \omega _0^2 \int _{-\infty }^{\infty } \frac{\left( f_{-}(v)+f_{+}(v)\right) }{1-\frac{|k| v}{\omega }} \mathrm {d} v, \end{aligned}$$

where *c* is the speed of light in vacuum. In this case, bearing in mind that light waves are high frequency waves, we may Taylor expand the integrand in Eq. (36) for $v \ll \omega / k$ and make use of Eq. (21), to obtain the following DR

37 $$\begin{aligned} \omega ^{2}=k^{2} c^{2}+\omega _{0}^{2}\left[ 1+\phi _i(q) \frac{k^{2} \lambda _{D}^{2} \omega _{0}^{2}}{k^{2} c^{2}+\omega _{0}^{2}}\right] , \end{aligned}$$

while there is no damping in this case. We will not discuss Eq. (37) any further since, because of the term $k^2c^2$, the corresponding DRs are notoriously weakly sensitive to the distribution function.

## Thermal Doppler broadening

We discuss in this section a possible experimental setup, based on a spectroscopic method known as thermal Doppler broadening, that may serve to directly identify the three universality classes of superstatistical velocity distributions. The method allows constructing the velocity distribution of a gas given its absorption spectrum and is particularly useful in probing velocity distributions in astrophysical situations, such as interstellar gas clouds^60^, space and astrophysical plasmas^61^, solar flares^62^, etc.

The mechanism behind this technique is quite simple. It is based on the broadening of the spectral lines caused by the Doppler effect on the velocity distribution of a gas of emitting particles. In fact, the velocity of each particle produces a Doppler shift of spectral wavelengths such that a characteristic wavelength $\lambda _0$, for particles at rest in the observer’s reference frame, is shifted to $\lambda = \lambda _{0}\left( 1+v / c\right)$, where *v* is the velocity component along the line of sight in the reference frame of the observer. The velocity distribution *f*(*v*) of the emitting particles can be fully determined from (and has the same form as) the distribution $f(\lambda )$ of the spectral shifts, since one has $f\left( v\right) d v = f(\lambda ) d \lambda$.

For a classical gas in equilibrium, the velocity distribution is a Maxwellian (Gaussian) distribution, and the distribution of the spectral shifts $f(\lambda )$ has the same form. The same reasoning applies to the superstatistical velocity distributions (1). In this case, the corresponding distribution of the spectral shifts is given as

38 $$\begin{aligned} f(\lambda )= \frac{c}{\lambda _0} \cdot B\left[ \frac{c\left( \lambda - \lambda _{0}\right) }{\lambda _{0}} \right] , \end{aligned}$$

where *B*(*x*) is given by Eq. (1), with $f(\beta )$ corresponding to one of the three universality classes, i.e., Eq. (2), (5), or (7). It is standard practice to characterize the Doppler broadening by the full width at half maximum (FWHM), which in the Maxwellian case, reads as^63^

39 $$\begin{aligned} \frac{\Delta \lambda }{\lambda _0}= \left( \frac{8 T}{m c^{2}} \ln 2\right) ^{1 / 2}. \end{aligned}$$

It is a simple exercise to identify the signature of the superstatistical distributions on the FWHM. In the case of $\chi ^2$ superstatistics, the distribution of the spectral shifts is merely a power-law and the corresponding FWHM can be easily computed as

40 $$\begin{aligned} \left( \frac{\Delta \lambda }{\lambda _0} \right) _{\chi ^2} = \left[ \frac{8 T_0}{m c^{2}} \left( \frac{2^{\frac{2q-2}{q+1}}-1}{q-1} \right) \right] ^{1 / 2}. \end{aligned}$$

In the limit $q \rightarrow 1$, the term in the parentheses in Eq. (40) reduces to $\ln 2$ and Eq. (40) reduces to Eq. (39), expressed at the mean temperature $T_0$. Note that, if one adopts the parametrization commonly used in Tallis statistics, i.e., $\tilde{q} \equiv 1+ (2q-2)/(q+1)$ and $\tilde{\beta } \equiv (q+1) \beta _0/2$, Eq. (40) can be expressed in the language of NSM^64,65^ as

41 $$\begin{aligned} \frac{\Delta \lambda }{\lambda _0} = \left[ \frac{8 \tilde{T}}{m c^{2}} \ln _{\tilde{q}}2 \right] ^{1 / 2} \end{aligned}$$

where

42 $$\begin{aligned} \ln _q x := \frac{x^{1-q}-1}{1-q} \end{aligned}$$

is the *q*-logarithm and $\tilde{T} \equiv 1/\tilde{\beta }$. For the two other universality classes, namely the inverse-$\chi ^2$ and the log-normal superstatistics, we are not in position to obtain closed-form expressions for the FWHM since this requires the variables to be solved for in an essentially non-algebraic way. One may however estimate the corresponding FWHM numerically. For typical atomic masses and temperatures, one has

43 $$\begin{aligned} \frac{m c^2}{T_0} \sim 10^{12}, \end{aligned}$$

and $\Delta \lambda / \lambda _0$ is of the order of $10^{-6}$. In this case, for a Maxwellian distribution, one has using Eq. (39), $\Delta \lambda / \lambda _0 \approx 2.3548 \, 10^{-6}$. Table 1 shows the estimated values corresponding to the three universality classes of superstatistics for different values of *q*. One may see that the presence of temperature inhomogeneities tends to decrease the FWHM; a feature that can be attributed to the heavy tails characterizing nonequilibrium distributions. Note that, although the estimated values presented in Table 1 are meant as an illustration, deviations of this order can, in principle, be measured with the present experimental technology that enables low-uncertainty measurements of spectral lines. In fact, the present experimental technology of spectroscopy achieves signal-to-noise ratios that exceed^66,67^ $10^5$ and a resolution of frequency as small as^68,69^ $10^{-12}$.

**Table 1:** Estimated values of $\Delta \lambda / \lambda _0$ ($\times 10^{-6}$) corresponding to the three universality classes of supertatistics, for different values of $q\equiv \langle \beta ^2 \rangle / \beta _0^2$.

$\Delta \lambda / \lambda _0$ ($\times 10^{-6}$)	$q=1.1$	$q=1.3$	$q=1.5$
$\chi ^2$	2.3365	2.2990	2.2610
Inverse $\chi ^2$	2.3446	2.3331	2.3306
Log-normal	2.3428	2.3192	2.3030

Finally, it should be stressed that, in addition to the thermal broadening discussed here, there exist other mechanisms, of different origins, responsible for the broadening of the spectral line width. One should mention the *pressure broadening*, due to collisions, and the *quantum broadening*, arising fundamentally from the uncertainty principle. These effects are known to produce a Lorentzian profile, and are totally independent on the thermal broadening^70^. This independence allows expressing the spectral line profile as a convolution of the two profiles. Using the superstatistical profile (38) for the thermal broadening, one may construct the corresponding Voigt functions, i.e., the joint profiles accounting for both thermal Doppler and Lorentzian broadenings (see for instance Ref.^61^).

## Conclusions

Given the growing empirical evidence in favor of distributions different from those predicted by equilibrium statistical mechanics, it becomes increasingly important to better understand their origins in physical problems. The best methodological attitude to obtain a complete and reliable picture of their origin consists in employing a synergistic approach, combining theoretical knowledge with experimental studies, involving direct and indirect measurements. This paper addresses an example of indirect measurements; those associated with dispersion relations (DRs). We have studied, in a quite general context, the signature of nonequilibrium distributions in DRs, by considering distributions in the form of a superposition of distributions, i.e., *superstatistics*. We focused our attention on the three universality classes of superstatistics, namely $\chi ^2$, inverse-$\chi ^2$, and log-normal universality classes, that have strong experimental evidence^23–27,29–33^. The extension of our analysis to other forms of distributions is nevertheless straightforward. We discussed the general context of systems obeying a combination of the Schrödinger and Poisson equations and studied more closely the case of classical DRs for a pair plasma. We have also presented a possible experimental setup to directly observe these distributions using spectroscopic methods.

Our analysis sheds light on the universal effect produced by nonequilibrium distribution on DRs. In fact, as DRs generally depend only on the first moments of the distribution, they do not contain the full information about it, and the signature of the latter is merely indicative of the heavy tails that are characteristic of nonequilibrium distributions.

This study may help probing the occurrence of nonequilibrium distributions in a variety of media exhibiting similar elementary excitations such as plasmons, hybrid-phonon modes, or Bogoliubov excitations in Bose–Einstein condensates. Possible new prospects for future research consist in going beyond the linear regime and investigating the effect of nonequilibrium distributions on the dynamics of nonlinear structures such as solitons, shock waves, voids, etc., that may take place in various medias.
